# LC‐Triple Quadrupole MS Enables Sample Classification in *Viscum combreticola*: Detecting Subtle Photochemical Isomerization in Hydroxycinnamic Acid Derivatives

**DOI:** 10.1155/jamc/7032335

**Published:** 2026-08-24

**Authors:** Mailane Rose Sekgobela, Anza-Tshilidzi Ramabulana, Lutendo Michael Mathomu, Babra Moyo, Ntakadzeni Edwin Madala

**Affiliations:** ^1^ Department of Biochemistry and Microbiology, Faculty of Science, Engineering and Agriculture, University of Venda, Private Bag X5050, Thohoyandou 0950, South Africa, univen.ac.za; ^2^ Meat Science and Technology, Animal Production, Agricultural Research Council, Old Olifantsfontein Road Irene, Centurion 0950, South Africa, arc.agric.za; ^3^ Department of Food Science and Technology, Faculty of Science, Engineering and Agriculture, University of Venda, Private Bag X5050, Thohoyandou 0950, South Africa, univen.ac.za

**Keywords:** geometrical isomerization, hydroxycinnamic acids, metabolomics, multivariate statistics, triple quadrupole (QQQ), UV light

## Abstract

High‐resolution MS techniques such as time‐of‐flight (TOF)‐MS are preferred for putative metabolite identification due to their unprecedented high resolution and accuracy in metabolomics studies. However, exploring cost‐effective alternative instruments for routine analyses would be advantageous, particularly in laboratories where access to high‐resolution mass spectrometry (MS) is limited. This study investigated the ability of the LC‐QQQ‐MS instrument operating in Q3 scan mode in replicating results obtained from the qTOF‐MS. Methanolic extracts of non‐treated and UV‐treated samples of *Viscum combreticola* were used as model samples for the analysis. Multivariate statistical models including principal component analysis (PCA), hierarchical clustering analysis and volcano plots were employed to highlight metabolite differences between UV‐treated and non‐treated plant extracts. The findings of this study demonstrated that the LC‐QQQ‐MS is effective in distinguishing between the two contrasting extracts of *V. combreticola*. Several *cis* isomers of hydroxycinnamic acid containing molecules such as 3‐feruloylquinic acid, 4,5‐dicaffeoylquinic acid and 4‐*p*‐coumaroyl‐5‐caffeoylquinic acids that underwent geometrical isomerization following UV exposure were identified as differentiating molecules (biomarkers), consistent with previous findings using high‐resolution qTOF‐MS instruments. This study positions QQQ‐MS as a valuable tool for authenticating samples containing known metabolites, particularly when the identification of unknowns is not the primary objective and high‐resolution MS instruments are not accessible.

## 1. Introduction

The metabolomics field is growing significantly with the continuous development of advanced analytical techniques, especially mass spectrometry (MS) for profiling metabolites in plants and other biological samples [1, 2]. High‐resolution MS instruments are often preferred due to their enhanced accuracy, enabling more reliable identification of metabolites [3, 4]. In studies where the primary aim is to classify or distinguish samples rather than identify unknowns, low‐resolution instruments can still provide valuable information, particularly when combined with multivariate statistical tools [5, 6]. These tools allow differentiating samples based on metabolite profiles and can be represented visually through statistical tools, even without complete metabolite identification.

Isomerization is a common natural phenomenon, and this may further be promoted by excessive sunlight exposure of plants caused by climate change as plants may undergo dynamic shifts in their metabolomes, particularly those rich in hydroxycinnamic acids (HCAs) [7]. As a result, there is a growing need for cost‐effective and reliable techniques to differentiate between plant extracts from various environments, particularly for authentication purposes since changes in the metabolic profile of plants can affect their medicinal properties. UV exposure induces *trans–cis* isomerization [8, 9], resulting in the formation of new metabolites (*cis* isomers), whose biological activities are still largely unknown. Although *trans–cis* isomerization does not produce significant changes in mass spectra as the resulting isomers are isobars, liquid chromatography (LC) can be used to separate these isomers based on their different retention times [10, 11]. This separation can be highlighted through peak‐picking algorithms and further analysed with multivariate statistical techniques to highlight differences between samples. In this study, the LC‐QQQ‐MS in a Q3 scan mode was used to analyse non‐treated and UV‐treated methanolic extracts of *Viscum combreticola* samples to assess its capability in replicating results from a quadrupole time‐of‐flight mass spectrometry (qTOF‐MS) instrument in metabolomics studies. This comparison is particularly significant for authentication studies, as it assesses whether the LC‐QQQ‐MS can provide a cost‐effective and accessible alternative for routine analysis in laboratories where high‐resolution MS may not be readily available. Multivariate statistical tools including, principal component analysis (PCA), hierarchical clustering analysis and volcano plots were employed to infographically display metabolite differences between the two sample groups.

## 2. Materials and Methods

### 2.1. Plant Harvesting and Sample Preparation

Stems and leaves of *V. combreticola* were collected from *Combretum erythrophyllum* in Shakadza, Nwanedi, Limpopo, in South Africa. The plants were dried, and secondary metabolites were extracted following the method described in a previous study [12]. Briefly, 2 g of dried and ground plant material was extracted in 20 mL of 80% methanol (UHPLC grade, Romil SpS, Cambridge, UK) on a digital rotisserie tube rotator (Dlab Scientific, Beijing, China) at 70 rpm overnight. The crude extracts were subsequently centrifuged at 2739 × *g* using a benchtop fixed‐angle centrifuge (Thermo Fisher, Johannesburg, South Africa) for 15 min. For UV irradiation, 1 mL of the methanolic extracts were exposed to a shortwave UV lamp at 254 nm in a UV lightbox for 24 h. The samples were filtered into HPLC vials through 0.22 μm Nylon filters before QQQ‐MS analysis. Replicates (*n* = 7) were prepared from the same sample.

### 2.2. Ultrahigh‐Performance Liquid Chromatograph Triple Quadrupole Mass Spectrometer Analysis

*V. combreticola* extracts were analysed on an ultrahigh‐performance liquid chromatograph triple quadrupole mass spectrometer (LCMS‐8045 QQQ, Shimadzu Corporation, Kyoto, Japan). The chromatographic separation was done on a Shim‐pack Sceptor C_18_ (100 mm × 2.1 mm, 1.9 µm) (Shimadzu Corporation, Kyoto, Japan) column housed in a heated oven (50°C). Separation of extracts was done using a binary mobile phase gradient, which consisted of solvent A: 0.1% formic acid in Milli‐Q water (both HPLC grade, Merck, Darmstadt, Germany) and solvent B: 0.1% formic acid in methanol (UHPLC grade, Romil SpS, Cambridge, UK). For all extracts, an injection volume of 0.5 μL was used and the flow rate was set to 0.3 mL/min throughout a 53‐min binary gradient. The mobile phase composition of the binary gradient was initiated with 10% B over 3 min, then gradually increased from 10% to 60% B over 3‐40 min, followed by an increase to 90% B between 43 and 45 min. The gradient was returned to 10% B between 50 and 53 min to achieve column re‐equilibration. Analysis was performed in the negative (ESI) mode using a low resolution QQQ in the Q3 scan mode. The scan range was set as *m*/*z* 100‐1500 Da with a scan speed of 1428 µ/s and an event time of 1 s. The other MS conditions were set as follows: desolvation temperature: 526°C, heating gas flow rate: 10 L/h, desolvation gas flow: 550 L/h and the interface temperature was 300°C. Under the same chromatographic separation conditions and similar MS conditions (varied conditions are stated in the following), product ion scans were acquired to obtain MS/MS spectra of selected *m*/*z* (*m*/*z* 397.1, 515.1, 367.1, 499.1, 473.1, 563.2 and 353.1) presumed to be HCA derivatives. The MS/MS spectra were obtained using collision energy of 30 eV, scan speed of 909 µ/s and event time of 1 s. Putative metabolite annotation was carried out based on precursor ion *m*/*z* values and mass spectral data from the QQQ‐MS product ion scan of the target compounds.

### 2.3. Multivariate Statistics

After data acquisition, data was analysed on MetaboAnalyst using the method outlined by Chong et al. [13]. The raw data files were converted to the mzML format, and they were then utilized for multivariate analysis with MetaboAnalyst 5.0 (https://www.metaboanalyst.ca/), an open‐access web‐based for processing metabolomics data. Each feature was defined by *m*/*z*, retention time and peak intensity. Data processing included missing value estimation (no missing values were detected using the default method), data integrity verification (default), log transformation, normalization by sum and Pareto scaling of datasets. Isotope grouping was not performed; however, ions identified as biomarkers and suspected to be isotopes were manually grouped with their counterparts during identification. Following data normalization and processing, the data were visualised using PCA, hierarchical clustering analysis and volcano plot.

## 3. Results and Discussion

**FIGURE 1:**
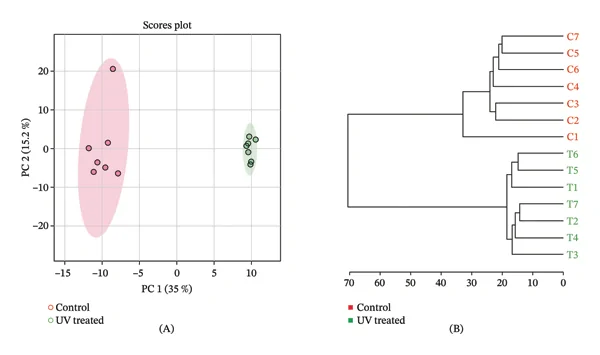
PCA score plot (A) comparing nontreated (red) and UV‐treated (green) features of *Viscum combreticola* extracts with a clear separation between the two groups implying a variation in their profiles with PC1 and PC2 scores accounting for 50.5% of the variation. Hierarchical clustering analysis (B) showed similarities within the nontreated and UV‐treated sample groups, respectively.

In this study, a low resolution QQQ mass spectrometer was used to analyse non‐treated and UV‐treated *V. combreticola* extracts to demonstrate the ability of this instrument in replicating results from the qTOF‐MS in metabolomics studies followed by data analysis using MVSA. Separation of the features from non‐treated and UV‐treated sample datasets using PCA as shown on (Figure 1A) into two clusters in non‐adjacent quadrants could imply differences in the two sample sets resulting from UV treatment. The score plot variance of PC1 and PC2 accounted for 50.5% of the total variance, as shown in Figure 1A. The clustering pattern shown in Figure 1A is supported by hierarchical clustering analysis as shown in Figure 1B, where two major nodes are observed, indicating differences between the UV‐treated extracts and the respective non‐treated samples [14].

**FIGURE 2:**
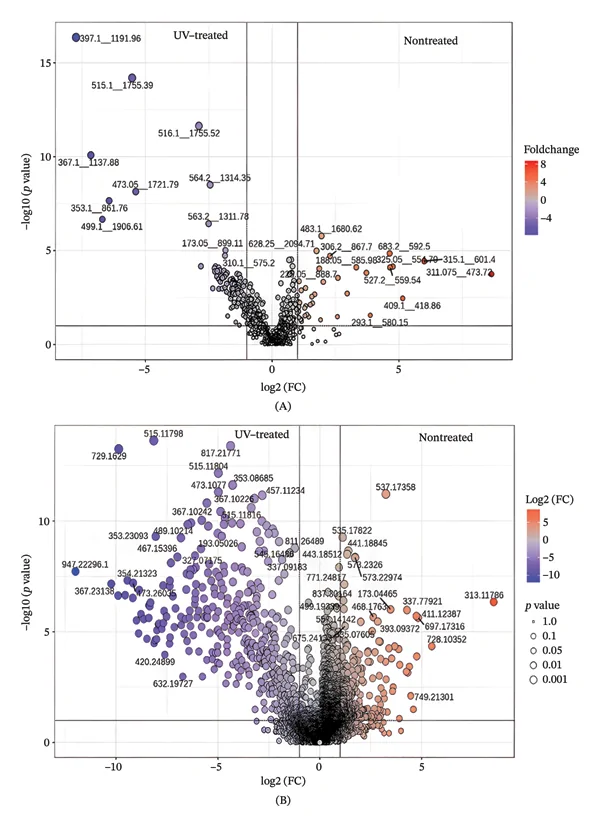
The volcano plot depicted statistically significant changes in UV‐induced ions compared to the relatively minor alterations observed in non‐UV‐induced ions in QQQ‐MS (A) and qTOF‐MS (B) instruments. Non‐UV‐induced features (red) were downregulated whereas UV‐induced ions (blue) were upregulated, respectively. The *x*‐axis represents the log_2_ fold change (FC), indicating the magnitude of change between conditions, while the *y*‐axis represents the ‐log10 (*p* value), indicating the statistical significance of the change. Low resolution mass spectrometry was used for this study and, therefore, isotopic peaks were manually grouped by checking masses that differed by 1 amu but sharing the same retention time.

**FIGURE 3:**
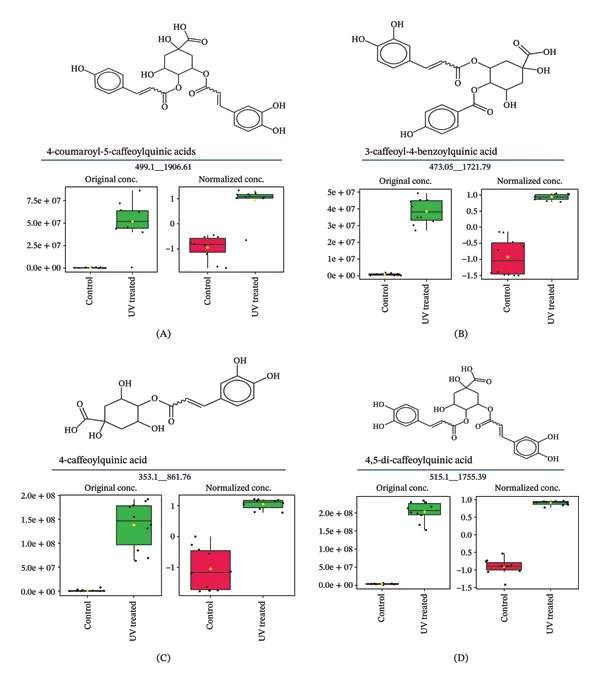
Box and whiskers plots illustrating the original and normalized relative peak abundances of the compounds *m*/*z* 499.1 (A), *m*/*z* 473.05 (B), *m*/*z* 353.1 (C) and *m*/*z* 515.1 (D) in the non‐treated and UV‐treated samples.

Based on the metabolite composition, the volcano plot shown on (Figure 2A) highlights an increased number of compounds in UV‐treated samples, particularly HCAs containing molecules at *m*/*z* 397.1, 515.1, 367.1, 499.1, 473.1, 563.2 and 353.1, indicating a notable response to UV light. A similar observation was also noticed with data obtained from the qTOF‐MS where compounds that were upregulated were mainly HCA derivatives (Figure 2B). This observation aligns with previous studies that reported the formation of new geometrical isomers of HCAs when plant extracts are exposed to UV light [12, 15]. The features representing metabolites that were upregulated according to Figure 2 were further explored using box and whisker plots (Figure 3 and S1) to show the ions’ relative peak intensities. Comparing the ions relative peak intensities in non‐treated and UV‐treated represented features, it can be seen that compounds at *m*/*z* 499.1, 473.05, 353.1, 515.1, 397.1, 367.1 and 563.2 exhibited a significant increase in relative peak intensities in UV‐treated samples compared to non‐treated samples, both in the original and normalized relative peak intensities data. This observation suggests that these compounds are *cis* isomers as they are amplified after UV treatment. While MVSA is a powerful tool for the identification of patterns and correlations, the integration of MS/MS and annotation of metabolites enhance the specificity and structural elucidation of these metabolites. Therefore, the capability of this instrument to differentiate between samples and replicate results from a qTOF‐MS offers researchers new opportunities to perform sample authentication in natural product research and metabolomics studies using significantly more affordable instrumentation.

**FIGURE 4:**
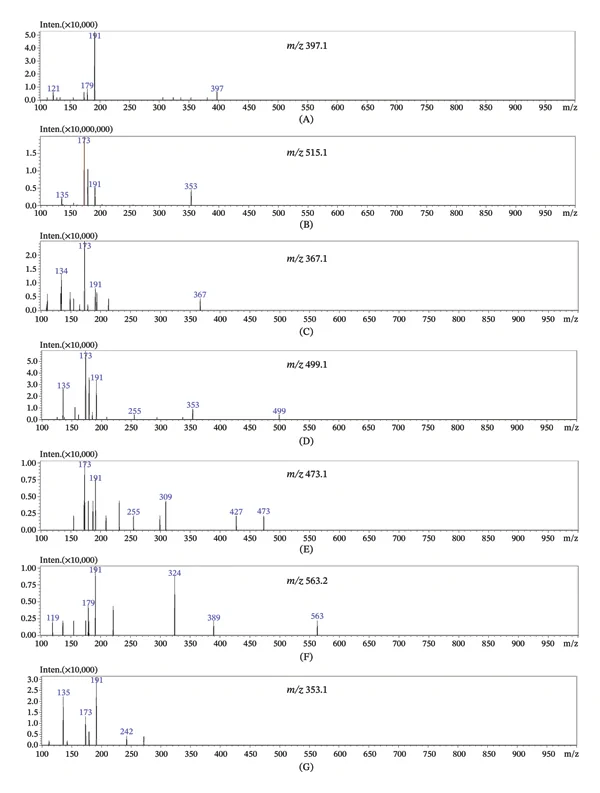
Product mass ion scan of (−) ESI‐QQQ MS/MS spectra showing fragmentations of molecular ions at *m*/*z* 397.1, 515.1, 367.1, 473.1, 499.1, 563.1 and 353.1 in spectra (A–G), respectively.

The HCA‐containing compounds observed on Figure 2 were further analysed by MS/MS product ion scan (Figure 4) to show their fragmentation patterns used to confirm the identity of these already known molecules. It is notable that fragmentation patterns obtained through product ion scan are very similar to qTOF‐MS, MS/MS data for the same compounds (Figure S2), supporting the potential of QQQ‐MS instruments in authenticating samples with a known chemical composition. Table 1 shows the targeted ions [*M* − *H*]^−^ at *m*/*z* 397.1, 515.1, 367.1, 473.1, 499.1, 563.3 and 353.1 that were putatively identified as 3‐sinapoylquinic acid, 4,5‐di‐caffeoylquinic acid, 3‐feruloylquinic acid, 3‐caffeoyl‐4‐benzoylquinic acid, 4‐coumaroyl‐5‐caffeoylquinic acid, caffeoylquinic acid derivative and 4‐caffeoylquinic acid, respectively, based on their fragmentation patterns. These identifications were done according to hierarchical scheme keys devised by Clifford et al. [16], where ion trap MS was employed. However, most of these compounds have been characterised extensively in other studies using qTOF‐MS instruments [12, 17, 18], demonstrating that the fragmentation behaviour observed in ion trap mass spectrometers can be replicated in other MS types, thereby allowing the identification of chlorogenic acids without the use of authentic standards [19]. Moreover, as observed in Table 1, the diagnostic ions of chlorogenic acids reported here are consistent with those previously reported using ion trap MS.

**TABLE 1:** Identified chlorogenic acids from QQQ mass spectrometric data showing molecular formula, fragmentation ions and isomeric compound names.

	*m*/*z*	rt	Molecular formula	Fragment ions	Compound name
1	397.1	19.87	C_18_H_22_O_10_	191, 173	3‐Sinapoylquinic acid
2	515.1	29.25	C_25_H_24_O_12_	353, 191, 173	4, 5‐Dicaffeoylquinic acid
3	367.1	18.96	C_17_H_20_O_9_	193, 191, 173	3‐Feruloylquinic acid
4	473.05	28.69	C_23_H_22_O_11_	191, 179	3‐Caffeoyl‐4‐benzoylquinic acid
5	353.1	14.36	C_16_H_18_O_9_	191, 173, 135	4‐Caffeoylquinic acid
6	499.1	31.77	C_25_H_24_O_11_	353, 337, 173	4‐Coumaroyl‐5‐caffeoylquinic acid
7	563.2	21.863	C_27_H_32_O_13_	191, 179, 135	Caffeoylquinic acid derivative

**FIGURE 5:**
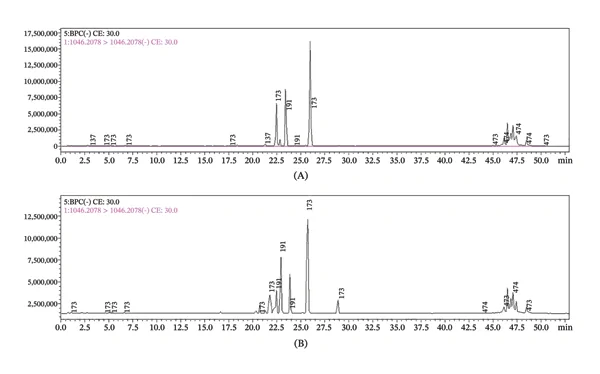
Extracted ion chromatograms showing geometrical isomers of caffeoyl‐hydroxylbenzoylquinic acid at *m*/*z* 473.1 in non‐treated (A) and UV‐treated (B) *Viscum combreticola* extracts.

Data from the qTOF‐MS that were initially used to identify these compounds are shown in Figure S2. In addition to a fragment ion at *m*/*z* 191 which is representative of the quinic acid (QA) moiety present in all these molecules, the presence of hydroxybenzoyl, caffeoyl, feruloyl and sinapoyl groups was confirmed by the presence of peaks at *m*/*z* 137, 179, 193 and 223, respectively (Figure 4). The intensity of the fragment ions of the QA moiety (*m*/*z* 191) and its dehydrated form (*m*/*z* 173) were used to identify the acylation position of the HCA moiety (s) on QA [16]. Moreover, regardless of the type of the HCA, the formation of geometrical isomers was indiscriminate. Figure 5A shows the extracted chromatogram of isomers of caffeoyl‐hydroxylbenzoylquinic acid obtained from the QQQ‐MS in non‐treated samples, whereas Figure 5B depicts an increased number of isomers of this compound attributed to geometrical isomerization that occurs in the presence of UV light. This observation aligns with findings from previous studies using high‐resolution MS [12, 20, 21], therefore highlighting the potential of the QQQ‐MS for authentication purposes. These studies reported that changes in metabolite composition of plants caused by light exposure contributes towards the chemical diversification of chlorogenic acids. Extracted ion chromatograms from the qTOF‐MS data for caffeoyl‐hydroxylbenzoylquinic acid (Figure S3) exhibited a comparable chromatographic pattern to the QQQ‐MS data, further reinforcing the potential of the QQQ‐MS as a reliable and cost‐effective instrument for targeted sample authentication workflows. The diversification of *V. combreticola* extracts in response to UV irradiation was further observed for several HCA derivatives as shown on Figure S4–S9. The newly formed compounds post UV irradiation for the compounds of interest in this study are *cis* isomers of the existing HCA derivatives in non‐treated samples as they have been observed to exhibit the same fragmentation pattern and they have been extensively characterized in our previous study [12]. A molecule at *m*/*z* 563.2 [*M* − *H*]^−^ was also observed in the UV‐irradiated samples (Figure 2 and Figure S1). The presence of fragment ions at *m*/*z* 353, 191 and 173 indicates that this compound is a caffeoylquinic acid–containing molecule. However, its exact identity could not be determined. This finding further highlights the limitations of QQQ‐MS for untargeted analyses. Nevertheless, as demonstrated here, QQQ‐MS can still be partially informative, as inherited structural moieties can be inferred from the presence of diagnostic fragment ions, as exemplified by this molecule at *m*/*z* 563.2. The different retention times for annotated compounds in Table 1 and Figure 4 provide further confirmation of structural differences in different isomers of chlorogenic acids annotated in this study. This highlights the ability of the QQQ‐MS instrument to distinguish metabolite markers in UV‐irradiated samples, similar to the findings reported by Moyo et al. [12] using the qTOF‐MS instrument.

## 4. Conclusion

This study explored the use of QQQ‐MS as a cost‐effective alternative in metabolomics studies. The findings of this study demonstrated that QQQ‐MS, despite its limitations in resolution, can effectively analyse complex samples and detect relevant markers related to UV exposure, offering comparable results to more sophisticated techniques like the UHPLC‐qTOF‐MS. These findings demonstrated that the QQQ‐MS can reliably authenticate samples containing known metabolites, making it a valuable tool for routine metabolomics analysis where targeted profiling is the primary focus. Nonetheless, high‐resolution MS techniques remain indispensable for comprehensive untargeted metabolomics that requires the identification of unknown compounds. The results presented here further reinforce the observation that cinnamic acid–containing molecules are highly susceptible to UV‐light exposure, undergoing photochemical isomerization. This phenomenon is expected to intensify as episodes of excessive sunlight associated with climate change become increasingly prevalent. Consequently, exploring cost‐effective analytical tools, such as the QQQ‐MS for screening applications, is imperative, particularly for compounds with well‐established diagnostic fragment ions, as exemplified by chlorogenic acids, which the biological consequences of their *cis* isomers remain unexplored.

## Author Contributions

Mailane Rose Sekgobela extracted the metabolites from plant samples, analysed the data and took part in writing the manuscript. Ntakadzeni Edwin Madala and Anza‐Tshilidzi Ramabulana ran mass spectrometry and wrote the manuscript. Babra Moyo took part in supervision and writing the manuscript. Ntakadzeni Edwin Madala and Lutendo Michael Mathomu conceived the study.

## Funding

No funding was received for this manuscript.

## Disclosure

All the authors have read and approved the final manuscript.

## Conflicts of Interest

The authors declare no conflicts of interest.

## Supporting Information

Additional supporting information can be found online in the Supporting Information section.

## Supporting information


**Supporting Information** Figure S1. Box and whisker plots showing relative abundance of metabolites at *m*/*z* 367.1 (A), 397.1 (B) and at *m*/*z* 563.2 (C) in UV‐treated and non‐treated *Viscum combreticola* samples. Figure S2. ESI‐qTOF MS/MS spectra showing fragmentations of molecular ions at *m*/*z* 397.1, 515.1, 367.1, 499.1, 473.1, 563.2 and 353.1 in A–G, respectively. Figure S3. Extracted ion chromatograms of geometrical isomers of hydroxylbenzoyl caffeoyl‐quinic acid at *m*/*z* 473.109 in non‐treated (A) and UV‐treated (B) samples obtained from the qTOF‐MS. Figure S4. Extracted ion chromatograms showing geometrical isomers of sinapoylquinic acid at *m*/*z* 397.1 in non‐treated (A) and UV‐treated (B) samples. Figure S5. Extracted ion chromatograms showing geometrical isomers of di‐caffeoylquinic acid at *m*/*z* 515.1 in non‐treated (A) and UV‐treated (B) samples. Figure S6. Extracted ion chromatograms showing geometrical isomers of feruloylquinic acid at *m*/*z* 367.1 in non‐treated (A) and UV‐treated (B) sample. Figure S7. Extracted ion chromatograms showing geometrical isomers of coumaroyl‐caffeoylquinic acids acid at *m*/*z* 499.1 in non‐treated (A) and UV‐treated (B) samples. Figure S8. Extracted ion chromatograms showing geometrical isomers of hydroxycinnamic acids containing compound at *m*/*z* 563.2 in non‐treated (A) and UV‐treated (B) samples. Figure S9. Extracted ion chromatograms showing geometrical isomers of caffeoylquinic acid at *m*/*z* 353.1 in non‐treated (A) and UV‐treated (B) samples.

## Data Availability

The data that support the findings of this study are available from the corresponding authors upon reasonable request.
