# Supplementary material for: LC‐Triple Quadrupole MS Enables Sample Classification in Viscum combreticola: Detecting Subtle Photochemical Isomerization in Hydroxycinnamic Acid Derivatives
Source: J Anal Methods Chem. 2026 Aug 24;2026:7032335. doi: 10.1155/jamc/7032335 (PMC13501164; doi:10.1155/jamc/7032335)
Supplement: Supplementary file 1 [file JAMC-2026-7032335-s001.docx]

**Supplementary information**

**UHPLC-qTOF-MS method**

The analysis of *V. combreticola* non-treated and UV-treated extracts was performed on a Shimadzu 9030 UHPLC-q-TOF-MS (Shimadzu Corporation, Kyoto, Japan). Chromatographic separation was achieved by injecting 5 µL of the sample onto a Shim-pack Scepter C_18_ column (100 × 2.1 mm, 1.9 µm) (Shimadzu Corporation, Kyoto, Japan), maintained at 55°C, with a binary mobile phase gradient at a flow rate of 0.3 mL min^-1^. Mobile phase A consisted of 0.1% (*v/v*) formic acid in ultrapure water, while mobile phase B comprised 0.1% (*v/v*) formic acid in methanol. The gradient started at 10% mobile phase B for 0-3 min, gradually increased to 60% to 40 min, this composition was maintained from 40 to 43 min, and further increased to 90% between 45 and 49 min. The gradient was then returned to 10% mobile phase B at 50 min and maintained until 53 min. Mass spectral analysis was performed using a high-resolution q-TOF mass spectrometer equipped with an electrospray ionization (ESI) interface in negative mode. Instrument settings included an interface voltage of 4.0 kV, interface temperature of 300°C, nebulization and dry gas flow at 3 L min^-1^, heat block temperature of 400°C, DL temperature of 280°C, detector voltage of 1.8 kV, and flight tube temperature of 42°C. Sodium iodide was used as a calibration solution to ensure accurate mass measurements. Ions in the *m/z* range of 100 to 1000 Da with intensities above 5000 were analyzed. MS^1^ and MS^2^ spectra were acquired simultaneously using data-dependent acquisition mode, with a collision energy of 30 eV and a spread of 5, with argon as the collision gas.

**Supplementary figures**

**Figure S1**. Box and whisker plots showing relative abundance of metabolites at *m/z* 367.1 (A), 397.1 (B) and at *m/z* 563.2 (C) in UV-treated and non-treated *Viscum combreticola* samples.

**Figure S2.** ESI-qTOF MS/MS spectra showing fragmentations of molecular ions at *m/z* 397.1, 515.1, 367.1, 499.1, 473.1, 563.2 and 353.1 in A, B, C, D, E, F and G, respectively.

**Figure S3**. Extracted ion chromatograms of geometrical isomers of hydroxylbenzoyl caffeoyl-quinic acid at *m/z* 473.109 in non-treated (A) and UV treated (B) samples obtained from the qTOF.

**Figure S4**. Extracted ion chromatograms showing geometrical isomers of sinapoylquinic acid at *m/z* 397.1 in non-treated (A) and UV treated (B) samples.

**Figure S5**. Extracted ion chromatograms showing geometrical isomers of di-caffeoylquinic acid at *m/z* 515.1 in non-treated (A) and UV treated (B) samples.

**Figure S6**. Extracted ion chromatograms showing geometrical isomers of feruloylquinic acid at *m/z* 367.1 in non-treated (A) and UV treated (B) sample.

**Figure S7**. Extracted ion chromatograms showing geometrical isomers of coumaroyl-caffeoylquinic acids acid at *m/z* 499.1 in non-treated (A) and UV treated (B) samples.

**Figure S8**. Extracted ion chromatograms showing geometrical isomers of hydroxycinnamic acids containing compound at *m/z* 563.2 in non-treated (A) and UV treated (B) samples.

**Figure S9**. Extracted ion chromatograms showing geometrical isomers of caffeoylquinic acid at *m/z* 353.1 in non-treated (A) and UV treated (B) samples.
